# Supplementary material for: A fault-intrusion-tolerant system and deadline-aware algorithm for scheduling scientific workflow in the cloud
Source: PeerJ Comput Sci. 2021 Nov 2;7:e747. doi: 10.7717/peerj-cs.747 (PMC8576554; doi:10.7717/peerj-cs.747)
Supplement: Supplemental Information 6 [file peerj-cs-07-747-s006.zip › Myproject1.1/docs/org/cloudbus/cloudsim/network/datacenter/AppCloudlet.html]

AppCloudlet


JavaScript is disabled on your browser.


- Overview
- Package
- Class
- Use
- Tree
- Deprecated
- Index
- Help

- Prev Class
- Next Class

- Frames
- No Frames

- All Classes

- Summary:
- Nested |
- Field |
- Constr |
- Method

- Detail:
- Field |
- Constr |
- Method


org.cloudbus.cloudsim.network.datacenter

## Class AppCloudlet

- java.lang.Object
- - org.cloudbus.cloudsim.network.datacenter.AppCloudlet

- Direct Known Subclasses:
  :   TestBagofTaskApp, WorkflowApp

  ---

    

  ```
  public class AppCloudlet
  extends java.lang.Object
  ```

  AppCloudlet class represents an application which user submit for execution within datacenter. It
  consist of several networkClouds.
  Please refer to following publication for more details:
  Saurabh Kumar Garg and Rajkumar Buyya, NetworkCloudSim: Modelling Parallel Applications in Cloud
  Simulations, Proceedings of the 4th IEEE/ACM International Conference on Utility and Cloud
  Computing (UCC 2011, IEEE CS Press, USA), Melbourne, Australia, December 5-7, 2011.

  Since:
  :   CloudSim Toolkit 1.0

- - ### Field Summary

    Fields

    | Modifier and Type | Field and Description |
    | --- | --- |
    | `double` | `accuracy` |
    | `static int` | `APP_MC` |
    | `static int` | `APP_Workflow` |
    | `int` | `appID` |
    | `java.util.ArrayList<NetworkCloudlet>` | `clist` |
    | `double` | `deadline` |
    | `double` | `exeTime` |
    | `int` | `numbervm` |
    | `int` | `requestclass` |
    | `int` | `type` |
    | `int` | `userId` |
  - ### Constructor Summary

    Constructors

    Constructor and Description

    `AppCloudlet(int type,
    int appID,
    double deadline,
    int numbervm,
    int userId)`
  - ### Method Summary

    Methods

    | Modifier and Type | Method and Description |
    | --- | --- |
    | `void` | `createCloudletList(java.util.List<java.lang.Integer> vmIdList)` An example of creating APPcloudlet |

    - ### Methods inherited from class java.lang.Object

      `clone, equals, finalize, getClass, hashCode, notify, notifyAll, toString, wait, wait, wait`

- - ### Field Detail


    - #### APP\_MC

      ```
      public static final int APP_MC
      ```

      See Also:
      :   Constant Field Values


    - #### APP\_Workflow

      ```
      public static final int APP_Workflow
      ```

      See Also:
      :   Constant Field Values


    - #### type

      ```
      public int type
      ```


    - #### appID

      ```
      public int appID
      ```


    - #### clist

      ```
      public java.util.ArrayList<NetworkCloudlet> clist
      ```


    - #### deadline

      ```
      public double deadline
      ```


    - #### accuracy

      ```
      public double accuracy
      ```


    - #### numbervm

      ```
      public int numbervm
      ```


    - #### userId

      ```
      public int userId
      ```


    - #### exeTime

      ```
      public double exeTime
      ```


    - #### requestclass

      ```
      public int requestclass
      ```
  - ### Constructor Detail


    - #### AppCloudlet

      ```
      public AppCloudlet(int type,
                 int appID,
                 double deadline,
                 int numbervm,
                 int userId)
      ```
  - ### Method Detail


    - #### createCloudletList

      ```
      public void createCloudletList(java.util.List<java.lang.Integer> vmIdList)
      ```

      An example of creating APPcloudlet

      Parameters:
      :   `vmIdList` - VMs where Cloudlet will be executed


- Overview
- Package
- Class
- Use
- Tree
- Deprecated
- Index
- Help

- Prev Class
- Next Class

- Frames
- No Frames

- All Classes

- Summary:
- Nested |
- Field |
- Constr |
- Method

- Detail:
- Field |
- Constr |
- Method
